# Friendly Kisses Can Be Deadly: *Capnocytophaga canimorsus* Bacteremia in an Asplenic Patient Exposed to Canine Saliva

**DOI:** 10.1155/2023/6618341

**Published:** 2023-12-20

**Authors:** Christina Rubio, Jared Miller, Tomasz Zrodlowski, Susanti Ie

**Affiliations:** ^1^Liberty University College of Osteopathic Medicine, USA; ^2^Carilion Clinic, Roanoke, USA

## Abstract

The differential diagnosis for febrile asplenic patients must always include opportunistic infections. *Capnocytophaga canimorsus* is one such infection. In this report, we discuss the case of a 73-year-old woman with a medical history significant for splenectomy for splenic sarcoma with prophylactic vaccination for pneumococcus who presented with rigors, emesis, and abdominal pain. Initial vital signs were 39.6°C (103.3°F), 166/70 mmHg, 92 bpm, and 95% SpO_2_ on room air. A physical examination revealed mild epigastric tenderness. Initial labs and imaging were unremarkable. Eight hours after the presentation, she became hypotensive. Repeat labs revealed leukopenia with 51% bands, hemoglobin 11.0 g/dL down from 13.9 g/dL, platelets 74 K/*μ*L trending down to 15 K/*μ*L, PT 23.5 sec., aPTT 60.3 sec., D-dimer greater than 20 *μ*g/mL, fibrinogen 190 mg/dL, LDH 1515 IU/L, haptoglobin less than 20 mg/dL, and creatinine 1.84 mg/dL. A peripheral smear showed schistocytes. Blood cultures identified gram-negative rods and *Capnocytophaga canimorsus*. After further questioning, she recalled her dog licking an abrasion on her left index finger. Four days after the presentation, she developed a purpuric rash on her bilateral hands and feet with areas of Nikolsky's negative bullae along the dorsum of her left foot. She also developed acute renal failure requiring renal replacement therapy and hemodialysis. *Capnocytophaga canimorsus* is an encapsulated facultative anaerobic gram-negative bacillus. Infection can result in bacteremia and sepsis and carries a high mortality rate, even with treatment. Those with hyposplenism/asplenia are particularly susceptible to infection and can deteriorate quickly, as seen in this case. Although this infection is rare, our case highlights how all asplenic patients must be assessed and treated for encapsulated bacterial infections when presenting with an acute febrile illness, regardless of initial laboratory analysis.

## 1. Introduction

Capnocytophaga canimorsus is an encapsulated facultative anaerobic gram-negative bacillus found in canine and feline saliva. It is associated with infections in both immunocompetent and immunocompromised hosts and can result in bacteremia and sepsis [1]. Those with hyposplenism or asplenia are particularly susceptible to infection and can deteriorate quickly. Other risk factors include heavy alcohol use, dog bites, male sex, and age over 50 years [1–3]. This report details the case of a 73-year-old woman who presented with fever, rigors, emesis, and epigastric abdominal pain. Labs and imaging were unremarkable at presentation, but, as the patient's clinical disposition deteriorated, blood cultures revealed infection with *Capnocytophaga canimorsus* due to the licking of an open wound by her pet dog. In this report, we detail the importance of considering *Capnocytophaga canimorsus* in febrile immunocompromised or asplenic patients, as these infections carry a high mortality rate [1].

## 2. Case Presentation

A 73-year-old woman presented to the hospital with rigors, emesis, right upper quadrant pain, and epigastric pain. Days prior, the patient had been gardening and noted a skin tear on her left index finger. Past medical history was significant for Grave's disease, partial gastrectomy, partial pancreatectomy, and splenectomy as part of her treatment for splenic sarcoma. She was up-to-date on the pneumococcal vaccine but was unsure of her meningococcal vaccination status.

On admission, the patient was noted to have a temperature of 39.6°C (103.3°F). Her blood pressure was 166/70 mmHg, heart rate was 92 bpm, and oxygen saturation was 95% on room air. The physical examination was unremarkable except for mild epigastric tenderness. Biochemical tests were all normal including the liver profile and lactic acid level. Computed tomography angiography (CTA) of the chest, abdomen, and pelvis was unremarkable but demonstrated fluid overload with third spacing seen around the epigastrium. The right upper quadrant ultrasound was also unremarkable.

Within a few hours after the presentation, she developed hypotension requiring vasopressor support. Repeat tests revealed leukopenia with 51% bands, thrombocytopenia, elevated prothrombin time (PT) and activated partial thromboplastin clotting time (aPTT), hypofibrinogenemia, and a D-dimer level above 20 *μ*g/mL. Cultures were obtained, and broad-spectrum antibiotics were initiated.

**Figure 1:**
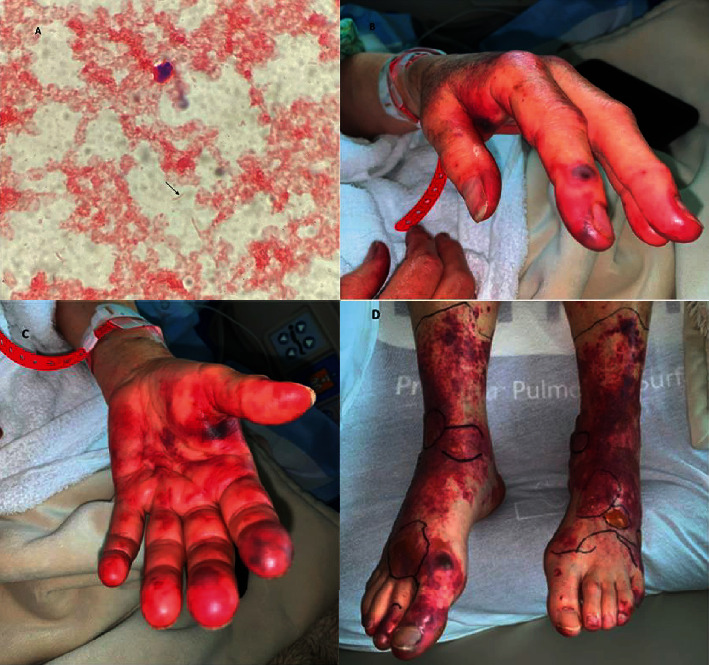
Clinical and pathological findings. (A) Peripheral blood smear showing gram-negative bacilli (arrow). (B, C) Upper extremity skin showing reddish skin discoloration. (D) Lower extremity skin changes with purpura fulminans.

A blood smear did show schistocytes. Circulating leukocytes consisted predominantly of mature segmented neutrophils and some band forms with increased cytoplasmic granularity and cytoplasmic vacuoles. Serological testing was negative for rheumatoid factor, antinuclear antibodies, and cardiolipin antibodies. The complement C3 level was decreased, but C4 was within normal limits. Ferritin was 3560 ng/mL, lactate dehydrogenase (LDH) was 1515 IU/L, haptoglobin was <20 mg/dL indicating hemolysis, and creatinine was 1.84 mg/dL. The dilute Russell viper venom test (DRVVT) was negative for lupus anticoagulant. A disintegrin and metalloproteinase with a thrombospondin type 1 motif, member 13, (ADAMTS-13) was within the normal range. Hepatitis B and C tests were negative. Molecular biology testing did not detect herpes simplex DNA virus or tick-borne disease (*Anaplasma*, *Babesia*, *Ehrlichia*, and *Borrelia*). Blood cultures showed gram-negative rods on day 1 and were negative for *Acinetobacter*, *Enterobacter*, *Proteus*, *E. coli*, *Klebsiella pneumonia*, *Klebsiella oxytoca*, and *Pseudomonas aeruginosa* by Verigene nucleic acid test. On day four, *Capnocytophaga canimorsus*, a capnophilic, facultative anaerobic, gram-negative rod, was identified (Figure 1(A)).

While in the ICU, the patient developed bilateral upper and lower extremity skin mottling without Nikolsky's sign but with areas of bullae along the dorsum of the left foot, approximately 2 × 1 cm in diameter, consistent with purpura fulminans (Figures 1(B)–1(D)). Antibiotics were switched to IV ampicillin/sulbactam after the speciation of the culprit pathogen. During her hospital course, she developed acute renal failure, requiring continuous renal replacement therapy. She also developed bilateral lower extremity deep venous thrombosis (DVT). The patient then started to clinically improve, her antibiotic therapy was continued, and she was transitioned to intermittent dialysis. She was then discharged home, where she underwent an additional 14 sessions of dialysis before having a full renal recovery. She has additionally required several debridements of her necrotic eschars associated with her purpura fulminans.

## 3. Discussion

In this case report, we discuss the effects of *Capnocytophaga*, a facultative anaerobic gram-negative bacillus, on the immunosuppressed and asplenic patient. There are two categories of *Capnocytophaga*: human-oral-associated *Capnocytophaga* and zoonotic *Capnocytophaga* (*canimorsus*, *canis*, and *cynodegmi*) [4, 5]. *Capnocytophaga* is associated with opportunistic infections not only in immunocompetent hosts but also in immunocompromised hosts and can result in bacteremia, sepsis, empyema, meningitis, chorioamnionitis, and wound infections [6]. Patients with hyposplenism, alcoholism, and cirrhosis are particularly susceptible to infection with *Capnocytophaga canimorsus* [4, 7]. In a review of cases of *C. canimorsus*, a history of exposure to dog bites, scratches, or licks is often present in patients with the infection. Although most cases have been noted in dogs, *C. canimorsus* can also occur in cats, so the diagnosis should be considered in patients who have had exposure to cats as well. Though *Capnocytophaga* infection is rarely seen in humans, *C. canimorsus* is the most common species of *Capnocytophaga* to cause disease in humans and an important consideration in patients presenting with bacteremia, as *C. canimorsus* carries a 10-30% mortality rate [4].

Diagnosis of *C. canimorsus* is challenging, as it can take 5-14 days to grow on blood cultures, and presentation is often nonspecific. Patients can present with fever, myalgias, chills, gastrointestinal symptoms, dyspnea, and altered mentation. Another possible finding, seen in this case, is purpura fulminans, which is a nonblanching purpuric rash that usually affects the extremities. Due to its insidious growth and vague presentation, achieving prompt diagnosis and treatment is difficult [8]. For this reason, a high index of suspicion should be maintained for patients with significant risk factors, as described above. Furthermore, clinicians should obtain cultures as soon as possible and start empiric antibiotics and supportive care promptly in any patients presenting with a sepsis-like picture to prevent further complications and mortality.

In regard to treatment, *Capnocytophaga* demonstrates susceptibility to beta-lactams, tetracyclines, erythromycin, clindamycin, fluoroquinolones, carbapenems, vancomycin, chloramphenicol, rifampin, and penicillin [1]. *C. canimorsus* specifically, however, rarely exhibits beta-lactamase production and is usually susceptible to penicillins, as demonstrated by our patient's clinical improvement in response to ampicillin therapy [4]. Length of treatment usually spans 14 to 21 days [8]. In conclusion, although human infection with *Capnocytophaga* is rare, it carries high mortality and can have a significant impact on the immunocompromised and should be considered in the appropriate clinical scenario.
